# Supplementary material for: Integrative Bioinformatics and Experimental Validation Establish CCNB1 as a Potential Biomarker for Diagnosis and Prognosis in Colorectal Cancer
Source: Curr Issues Mol Biol. 2025 Dec 9;47(12):1026. doi: 10.3390/cimb47121026 (PMC12731441; doi:10.3390/cimb47121026)
Supplement: Supplementary file 1 [file cimb-47-01026-s001.zip › caption.pdf]

current issues in molecular biology

MDPI

Table S1: 197 common DEGs in GSE74602, GSE103512, TCGA-COAD.

Table S2: GO enrichment of DEGs.

Table S3: KEGG enrichment of DEGs.

Table S4: Kaplan-Meier survival analysis of 16 genes.

Table S5: Univariate Cox analysis of 21 genes.

Figure S1: Volcano plots of DEGs between CRC and normal tissue from (a) GSE74602, (b) GSE103512, and (c) TCGA-COAD datasets. DEGs were defined by $|\log_2(\text{fold change})| > 1$ and *p-value* < 0.05. Upregulated genes are highlighted in red, downregulated genes in blue, and non-significant genes in gray.

Figure S2: Bubble plots displaying the top 5 enriched GO terms for each category, derived from (a) upregulated and (b) downregulated DEGs.

Figure S3: PPI network analysis (a) Visualization of the PPI network. Upregulated DEGs are shown in red and downregulated DEGs in blue. (b) PPI network showing the 17 hub genes marked in yellow.

Figure S4: ROC curve of the 17 hub genes.

Figure S5: Kaplan-Meier survival analysis of 8 prognostic genes assessed by the log-rank test.

Figure S6: Heatmap showing the expression of the top 10 marker genes across cell clusters in a CRC scRNA-seq dataset.

Figure S7: Violin plot of CCNB1 expression across cell clusters in a CRC scRNA-seq dataset.

Figure S8: Violin plots of S-phase and G2M-phase scores across cell clusters in a CRC scRNA-seq dataset.

Figure S9: Cell cycle analysis of SW480 cells by flow cytometry in the three groups: (a) control. (b) si-NC. (C) si-CCNB1.
