# Supplementary figures and images for: Integrative Bioinformatics and Experimental Validation Establish CCNB1 as a Potential Biomarker for Diagnosis and Prognosis in Colorectal Cancer
Source: Curr Issues Mol Biol. 2025 Dec 9;47(12):1026. doi: 10.3390/cimb47121026 (PMC12731441; doi:10.3390/cimb47121026)

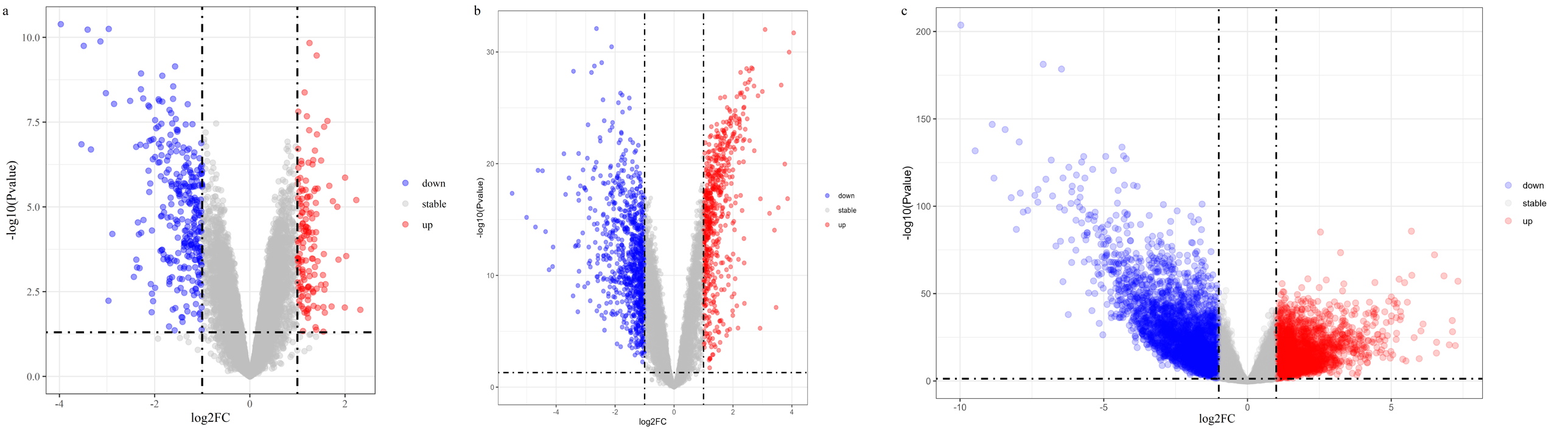

Supplement: Supplementary file 1 [file cimb-47-01026-s001.zip › Figure S1.png]

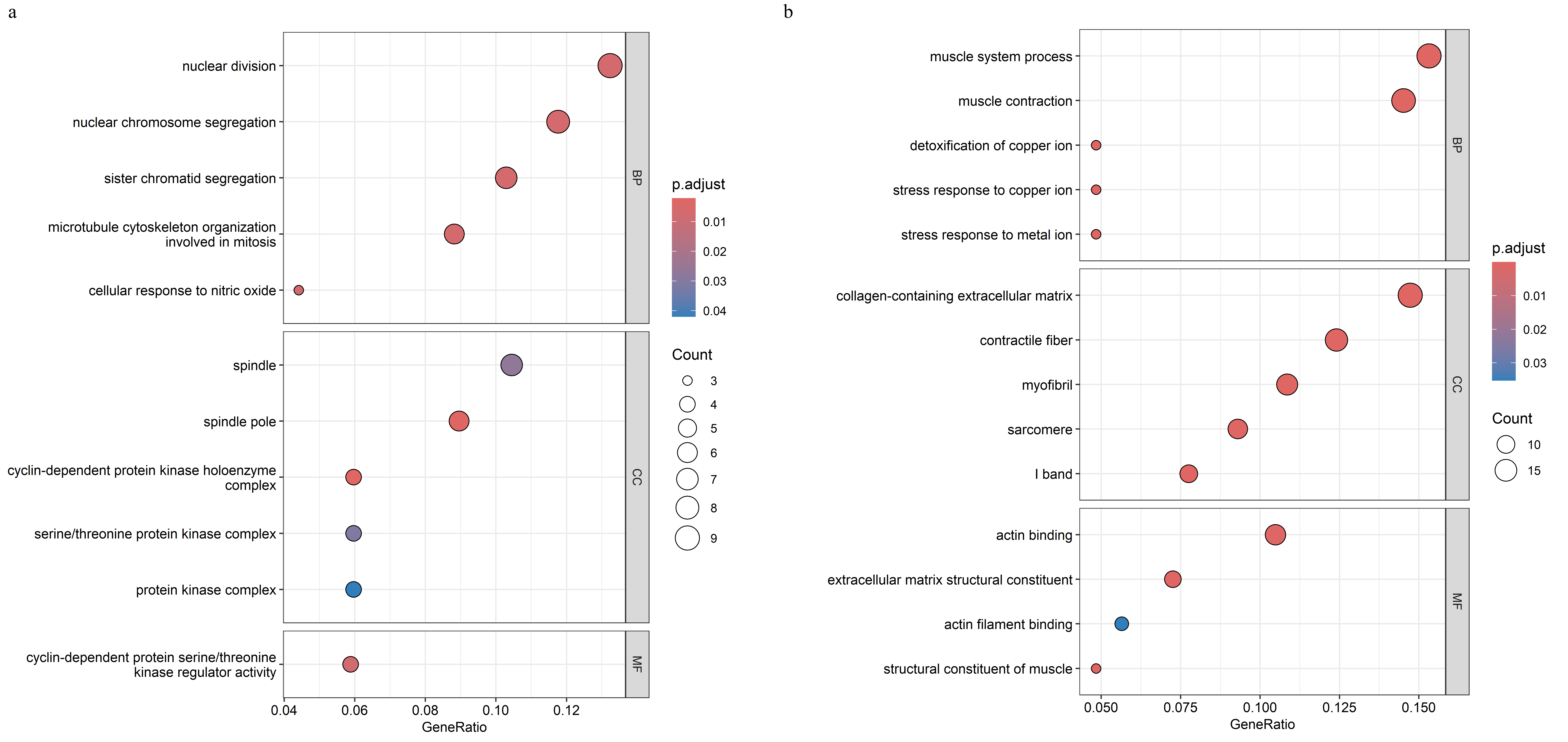

Supplement: Supplementary file 1 [file cimb-47-01026-s001.zip › Figure S2.png]

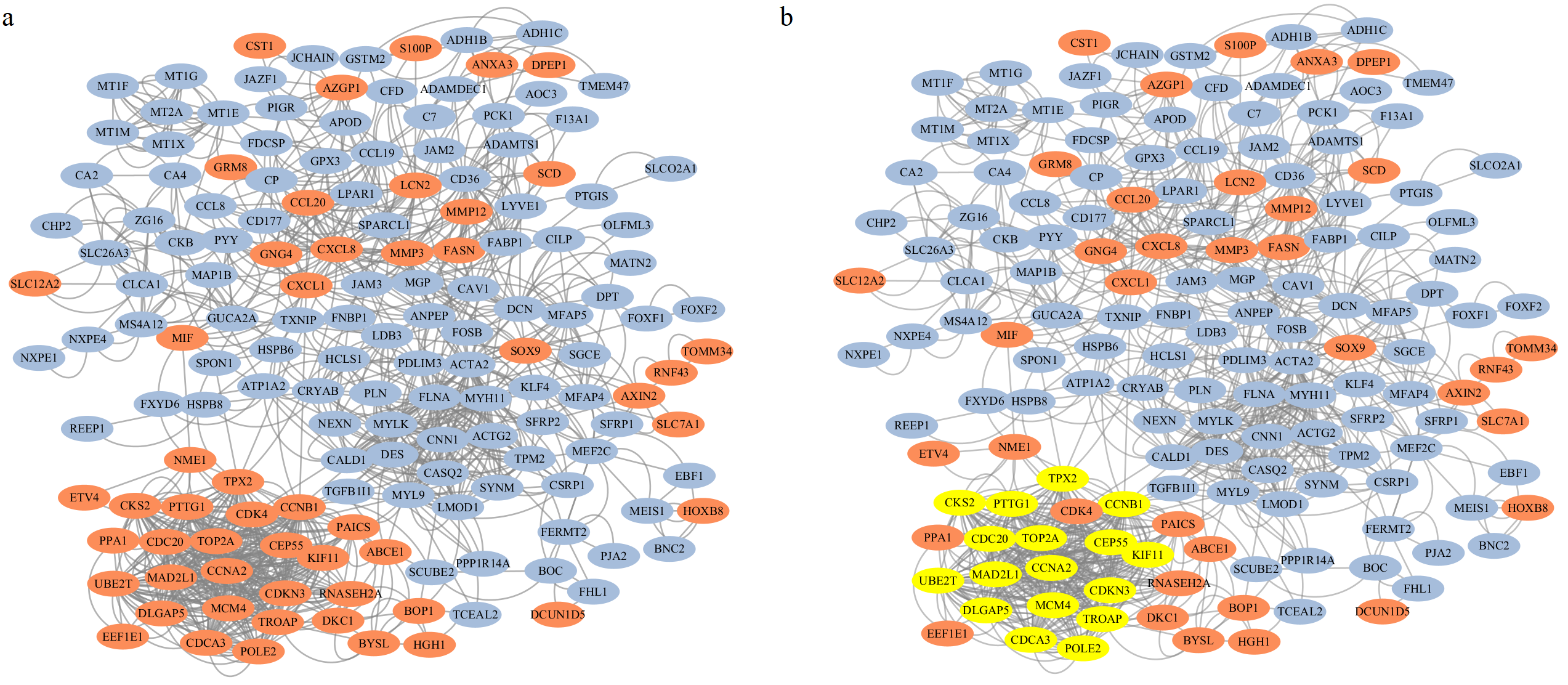

Supplement: Supplementary file 1 [file cimb-47-01026-s001.zip › Figure S3.png]

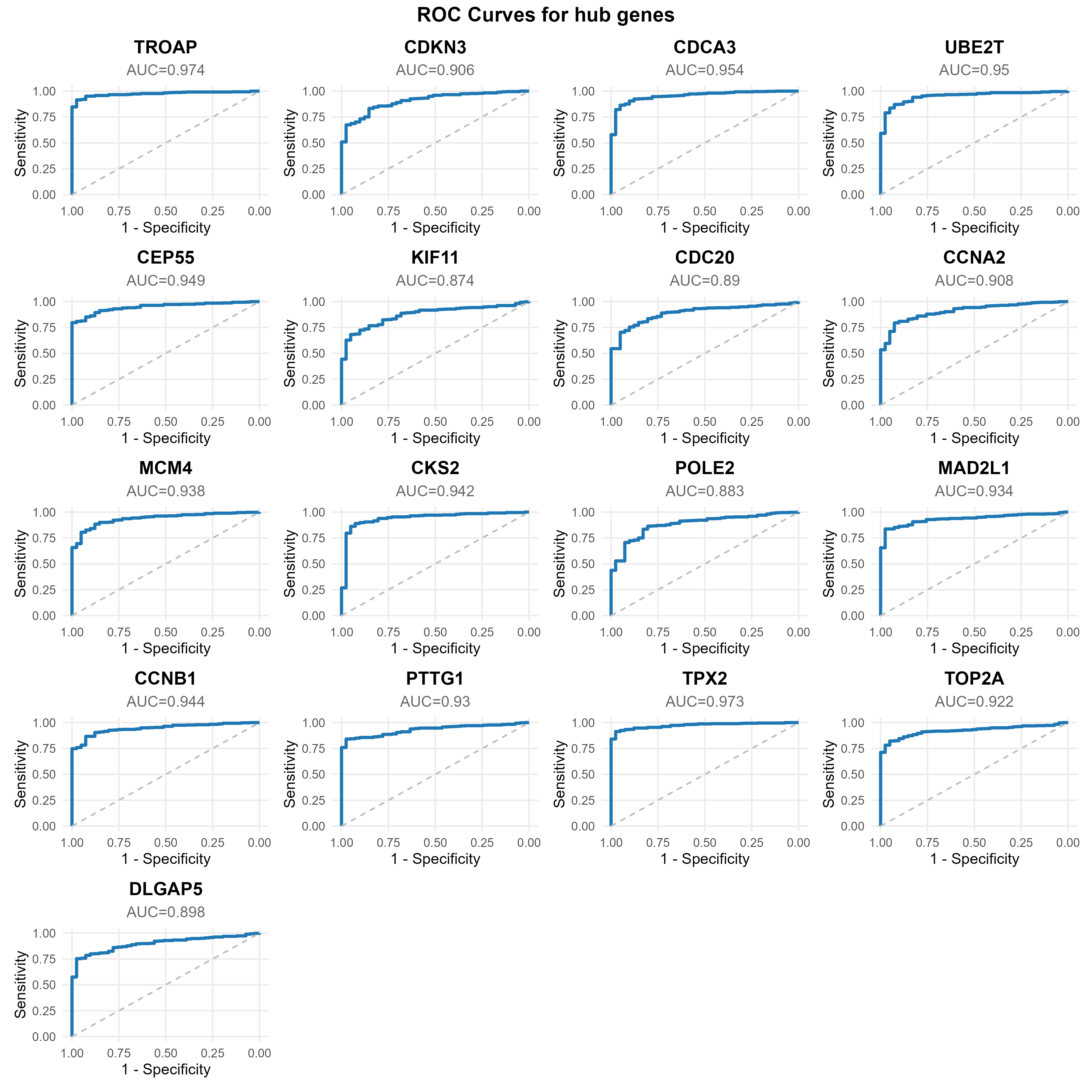

Supplement: Supplementary file 1 [file cimb-47-01026-s001.zip › Figure S4.png]

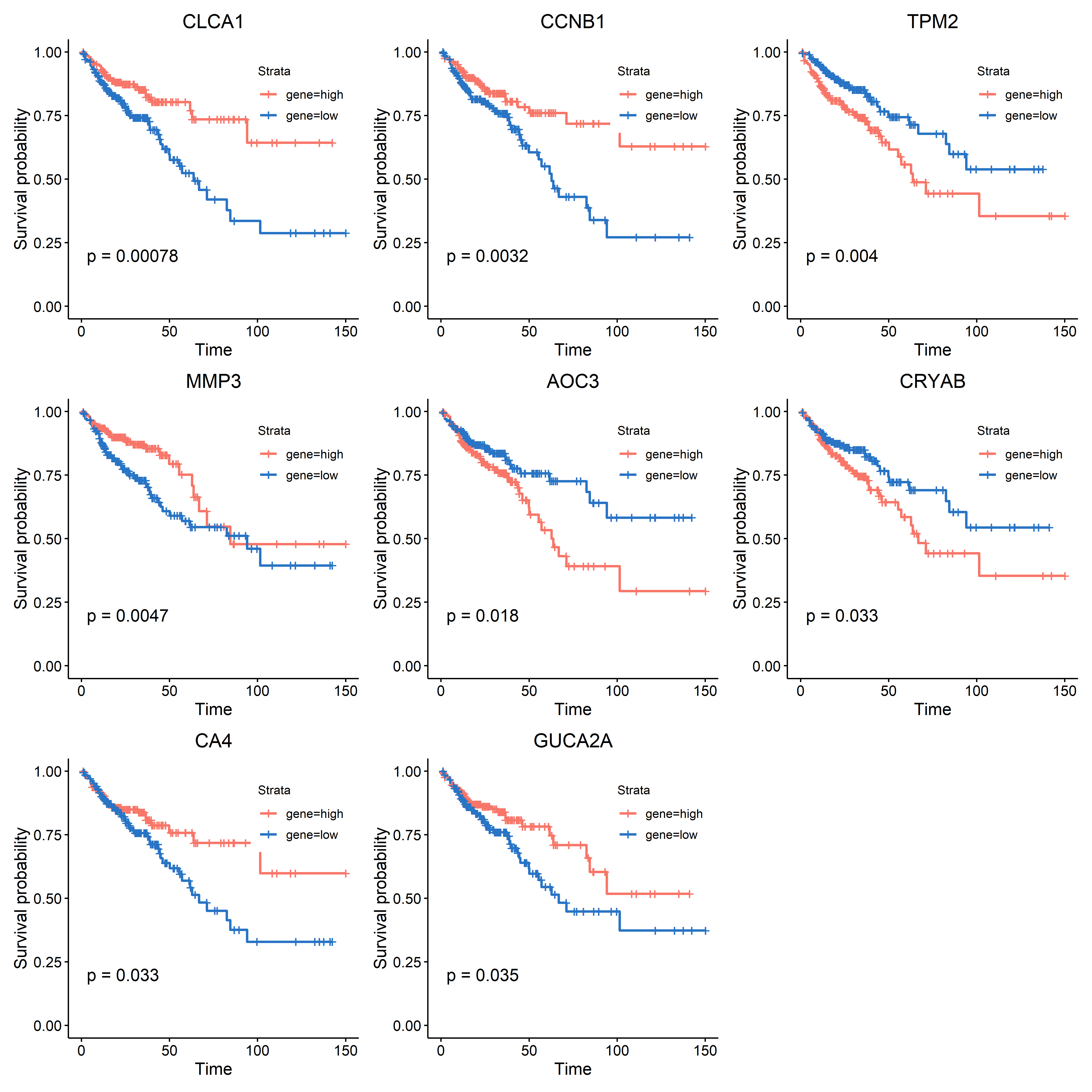

Supplement: Supplementary file 1 [file cimb-47-01026-s001.zip › Figure S5.png]

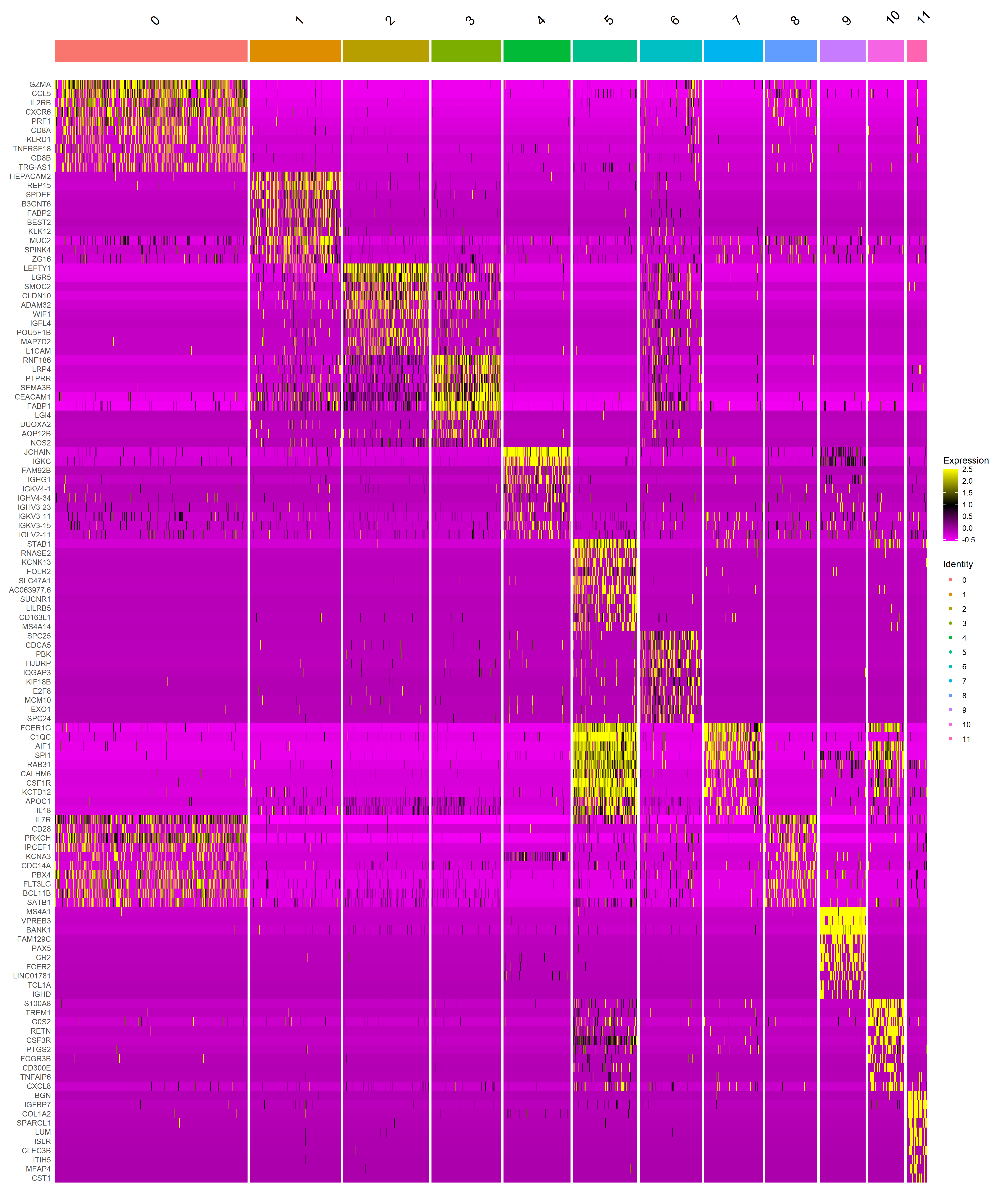

Supplement: Supplementary file 1 [file cimb-47-01026-s001.zip › Figure S6.png]

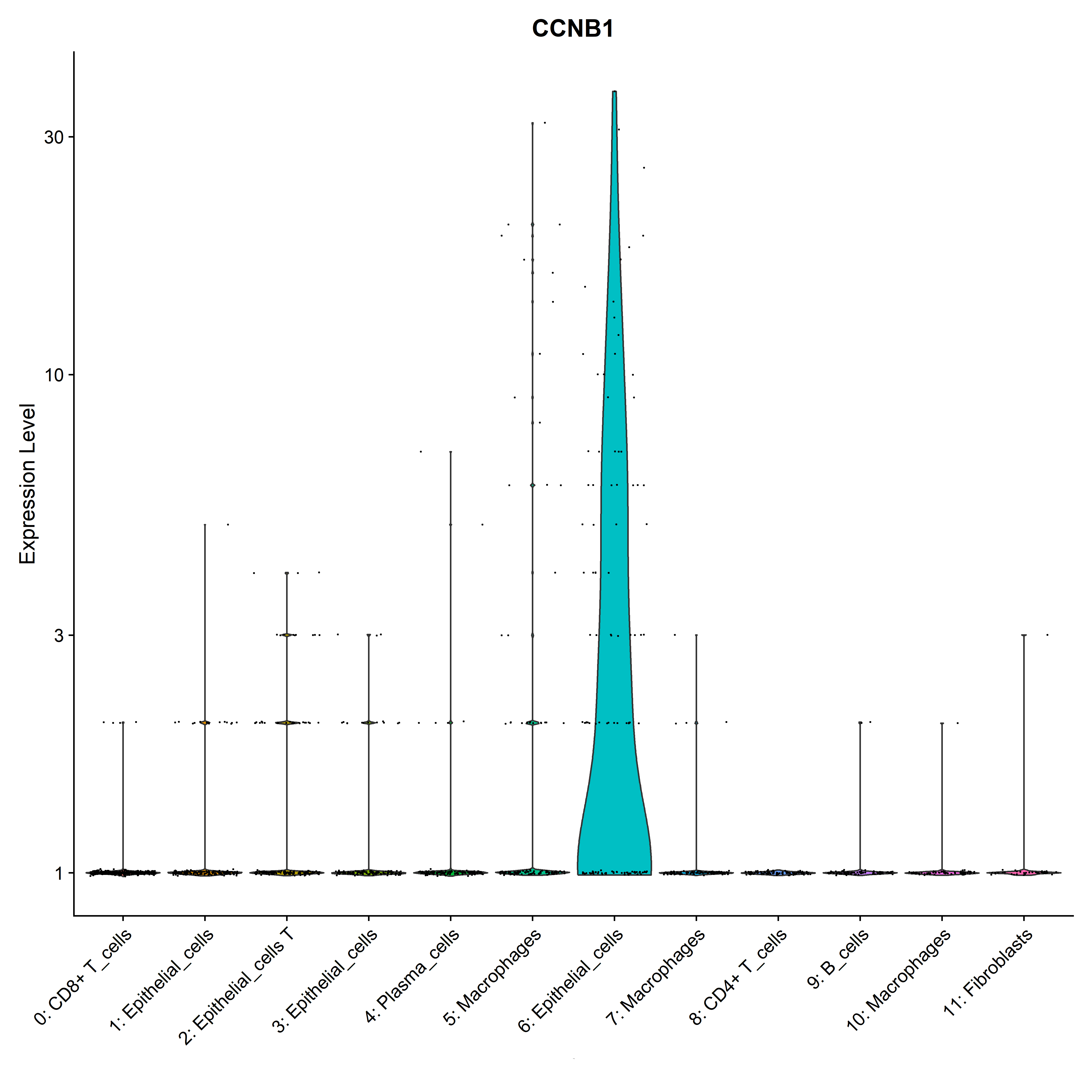

Supplement: Supplementary file 1 [file cimb-47-01026-s001.zip › Figure S7.png]

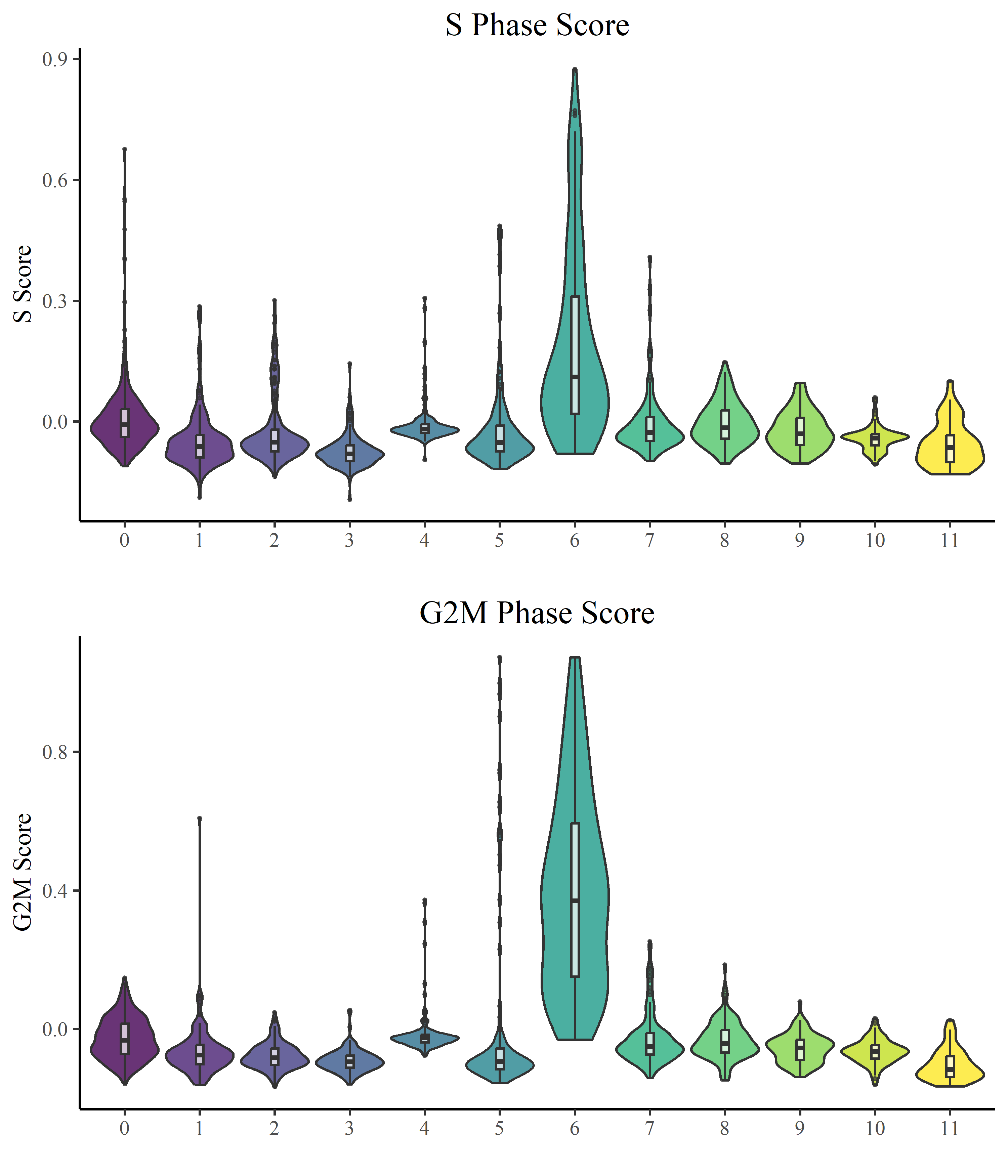

Supplement: Supplementary file 1 [file cimb-47-01026-s001.zip › Figure S8.png]

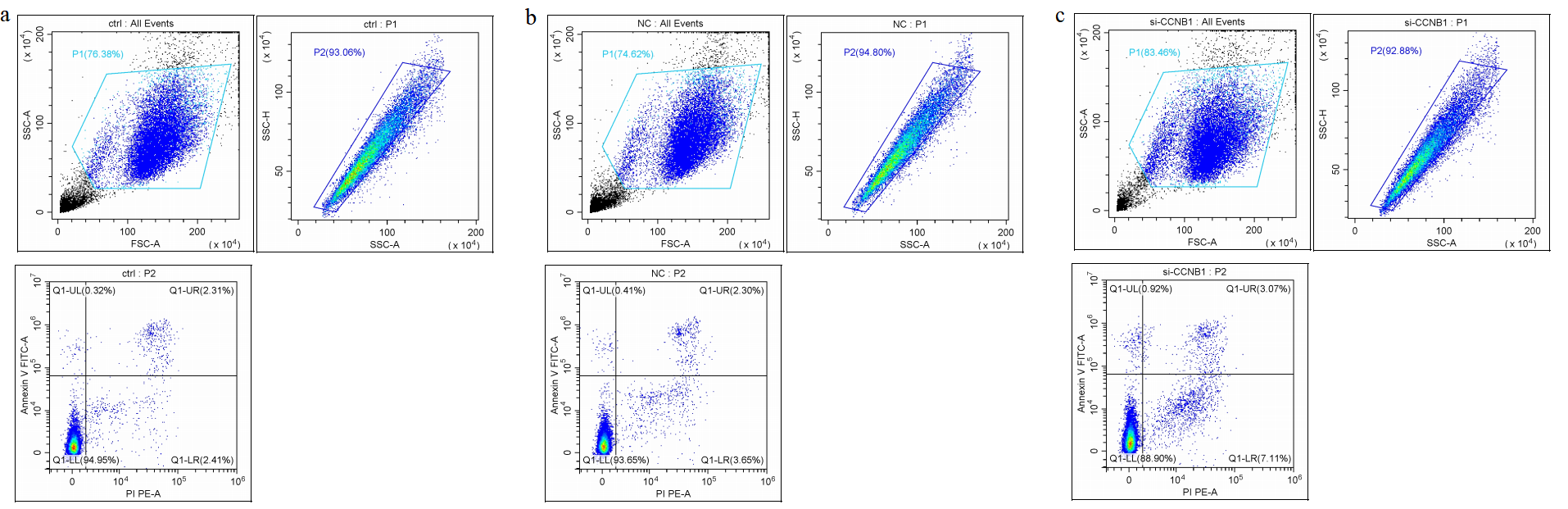

Supplement: Supplementary file 1 [file cimb-47-01026-s001.zip › Figure S9.png]
